# Autumnal Catarrh

**Published:** 1872-08

**Authors:** 


*Autumnal Catarrh, (Hay Fever), with three maps.* By MORRILL WYMAN, M. D. New York: Hurd & Houghton, 1872. Buffalo: Martin Taylor.

That a peculiar and distressing form of catarrh exists in the northern portions of North America during certain seasons of the year has come to be generally admitted, although, from being confined to certain localities, it has not fallen under the general observation of the profession.

This disease was first described in 1854, by Prof. Wyman in his lectures in the Medical School of Harvard University. Being a sufferer himself, Prof. Wyman has given this disease much careful thought and inquiry, and the result of his labors is embodied in the work now under consideration. The book comprises over one hundred and seventy pages, and treats of the affection in the following order:

General History, Local Symptoms, Constitutional Symptoms, Annual Course, Geographic and Chorographic Relations, Influence of place upon the disease, Causes of Paroxysms, Diagnosis, Prognosis, Treatment, and Illustrative Cases.

The large experience which Dr. Wyman has had with this disease, has made him competent to speak with authority upon the subject, and we do not hesitate to recommend the work to such of our readers who may wish for information in regard to it.

